# Midbrain cytotoxic T cells as a distinct neuropathological feature of progressive supranuclear palsy

**DOI:** 10.1093/brain/awaf135

**Published:** 2025-04-15

**Authors:** Blas Couto, Shelley L Forrest, Conor Fearon, Seojin Lee, Samantha Knott, Jun Li, Susan H Fox, Maria Carmela Tartaglia, Anthony E Lang, Gabor G Kovacs

**Affiliations:** Favaloro University Hospital, Institute of Cognitive and Translational Neuroscience (INCyT) Favaloro-INECO-CONICET, Buenos Aires CA1020, Argentina; Tanz Centre for Research in Neurodegenerative Diseases, University of Toronto, Toronto, Ontario M5T 0S8, Canada; Tanz Centre for Research in Neurodegenerative Diseases, University of Toronto, Toronto, Ontario M5T 0S8, Canada; Krembil Brain Institute, University Health Network, Toronto, Ontario M5T 0S8, Canada; Dublin Neurological Institute at the Mater Misericordiae University Hospital, Dublin D07 W7XF, Ireland; Edmond J. Safra Program in Parkinson’s Disease and the Morton and Gloria Shulman Movement Disorders Clinic, Toronto Western Hospital, Toronto, Ontario M5T 2S8, Canada; Tanz Centre for Research in Neurodegenerative Diseases, University of Toronto, Toronto, Ontario M5T 0S8, Canada; Tanz Centre for Research in Neurodegenerative Diseases, University of Toronto, Toronto, Ontario M5T 0S8, Canada; Tanz Centre for Research in Neurodegenerative Diseases, University of Toronto, Toronto, Ontario M5T 0S8, Canada; Edmond J. Safra Program in Parkinson’s Disease and the Morton and Gloria Shulman Movement Disorders Clinic, Toronto Western Hospital, Toronto, Ontario M5T 2S8, Canada; Rossy Centre for PSP, Toronto Western Hospital, Toronto, Ontario M5T 2S8, Canada; Division of Neurology, University of Toronto, Toronto, Ontario M5S 1A1, Canada; Tanz Centre for Research in Neurodegenerative Diseases, University of Toronto, Toronto, Ontario M5T 0S8, Canada; Krembil Brain Institute, University Health Network, Toronto, Ontario M5T 0S8, Canada; Rossy Centre for PSP, Toronto Western Hospital, Toronto, Ontario M5T 2S8, Canada; Division of Neurology, University of Toronto, Toronto, Ontario M5S 1A1, Canada; Tanz Centre for Research in Neurodegenerative Diseases, University of Toronto, Toronto, Ontario M5T 0S8, Canada; Edmond J. Safra Program in Parkinson’s Disease and the Morton and Gloria Shulman Movement Disorders Clinic, Toronto Western Hospital, Toronto, Ontario M5T 2S8, Canada; Rossy Centre for PSP, Toronto Western Hospital, Toronto, Ontario M5T 2S8, Canada; Division of Neurology, University of Toronto, Toronto, Ontario M5S 1A1, Canada; Tanz Centre for Research in Neurodegenerative Diseases, University of Toronto, Toronto, Ontario M5T 0S8, Canada; Krembil Brain Institute, University Health Network, Toronto, Ontario M5T 0S8, Canada; Edmond J. Safra Program in Parkinson’s Disease and the Morton and Gloria Shulman Movement Disorders Clinic, Toronto Western Hospital, Toronto, Ontario M5T 2S8, Canada; Rossy Centre for PSP, Toronto Western Hospital, Toronto, Ontario M5T 2S8, Canada; Division of Neurology, University of Toronto, Toronto, Ontario M5S 1A1, Canada; Department of Laboratory Medicine and Pathobiology, University of Toronto, Toronto, Ontario M5S 3K3, Canada; Laboratory Medicine Program, University Health Network, Toronto, Ontario M5G 2C4, Canada

**Keywords:** inflammation, neuropathology, progressive supranuclear palsy, tau, T-lymphocyte

## Abstract

Progressive supranuclear palsy (PSP) is a neurodegenerative disorder characterized by four-repeat (4R) tau protein deposition. The substantia nigra (SN) and midbrain tegmentum nuclei (MBT) are consistently affected. Lymphocyte infiltrates are scarce in the brains of patients with neurodegenerative diseases, although a few reports have described their presence in the α-synucleinopathy Parkinson's disease (PD).

To evaluate the cytotoxic T-cell response, serial sections spanning 120 μm of the SN were immunostained consecutively for phosphorylated tau (p-tau, AT8) or α-synuclein, cytotoxic T-cell marker and microglia marker HLA-DR. Sections were analysed with stereology software in 9 patients with PSP, 10 with PD and 6 healthy controls. We semiquantitatively scored CD8-positive cells in further brain regions.

CD8 lymphocyte cell counts and microglial activation in the SN were higher in PSP than PD and controls. Furthermore, T-cell/neuron contact was observed in PSP. In multivariate models, CD8 counts were not predicted by disease duration, younger age at death or the amount of p-tau pathology. The SN and midbrain tegmentum showed more CD8 cells than the cortex.

A more prominent nigral cytotoxic T-cell response in PSP than PD supports the suggestion that p-tau neuropathology in PSP might have potential relationships with autoimmune mechanisms.

## Teaser

Inflammation has been suggested to play a role in neurodegenerative disease. Couto *et al.* found increased numbers of cytotoxic T cells in the substantia nigra of patients with progressive supranuclear palsy (PSP) compared to Parkinson's disease, suggesting a potential autoimmune component in the pathogenesis of PSP.

## Introduction

Progressive supranuclear palsy (PSP) is a neurodegenerative disorder characterized by the deposition of predominantly four-repeat (4R) isoforms of tau in neurons and glial cells. The substantia nigra (SN), along with the midbrain tegmentum nuclei (MBT), the subthalamic nucleus (STN) and the globus pallidus (GP) are affected early in the disease progression.^1^

Microglial activation has been revealed in PSP,^2^ and inflammatory markers have been detected in blood^3^ and by neuroimaging, predicting progression.^4,5^ Higher transcription of interleukin-1β in the SN of patients with PSP compared with Alzheimer's disease (AD) has been reported.^6^ Controversial results exist regarding associations between symptom severity and peripheral cytokines or CSF-measured interleukins. One study found a correlation between PSP staging and peripheral IL-6^7^ and another showed a lack of correlation between CSF cytokines and PSP Rating Scale (PSPRS) scores.^8^

Importantly, lymphocytic cell infiltrates are characteristic of infectious and autoimmune encephalitides. T cells apposed to neurons can be seen in autoimmune processes.^9^ In contrast, neurodegenerative diseases are traditionally distinguished by a lack of inflammatory cell infiltrates. Although evidence of lymphocyte infiltrates in neurodegenerative diseases is scarce, previous studies have revealed a shift in CD8/CD4 ratio in blood and CSF of PSP patients.^10,11^ A recent report focusing on the frontal cortex showed a correlation between CD8 cells and phosphorylated-tau (p-tau) in frontotemporal-lobar degeneration (FTLD), potentially mediated by microglia and astroglial activation.^12^ In Parkinson's disease (PD), an α-synucleinopathy with Lewy-bodies, CD8 infiltration has been detected in the early stages of pathology.^13^

The goal of this study was to evaluate the presence of cytotoxic T-lymphocytes and their relation to microglial activation and tau pathology load, focusing on early vulnerable regions, the SN and MBT, in patients with PSP or PD and in non-diseased controls (CO).

## Material and methods

### Selection of cases

Post-mortem human brain samples were obtained from the University Health Network Neurodegenerative Disease Brain Collection (UHN-NBC). Autopsy tissue was collected with informed consent of patients or their relatives and approval of the local institutional review board (UHN Research Ethics Board Number 20-5258). We selected 19 patients (10 with PD, 9 with PSP) and 6 non-diseased controls (CO; Table 1). Neuropathological diagnosis followed the Lewy body disease consensus criteria^14^ and Braak stage of Lewy-related pathology,^15^ the Rainwater criteria for PSP^16^ and staging for PSP^1^ and argyrophilic grain disease (AGD).^17^ We screened for mixed pathologies as summarized in a recent review.^18^

**Table 1:** Clinical and pathological features of cases included in the study

Case	Age, years	Sex	Disease duration, years	PSP stage^1^	AGD stage^17^	Braak NFT stage^18^	Thal Aβ phase^19^	Braak LBD stage^14^ ^,15^	LATE-NC^21^	CAA
PSP-1	70	Female	9	5	0	II	0	0	0	−
PSP-2	72	Male	11	4	I	II	0	0	0	−
PSP-3	72	Male	11	5	0	I	0	0	0	−
PSP-4	77	Male	8	4	I	II	1	0	0	−
PSP-5	71	Male	6	3	0	II	0	0	0	−
PSP-6	73	Male	3	4	III	II	0	0	0	+
PSP-7	76	Male	5	3	I	II	0	0	0	−
PSP-8	63	Male	3	4	0	II	0	0	0	−
PSP-9	63	Male	5	3	0	I	1	0	0	−
LBD-1	56	Male	12	0	0	V	4	6	0	+
LBD-2	66	Male	11	0	0	II	1	4	0	−
LBD-3	85	Male	11	0	II	II	0	4	2	−
LBD-4	69	Male	6	0	0	IV	3	6	0	+
LBD-5	76	Male	8	0	0	IV	3	5	0	−
LBD-6	73	Male	20	0	0	II	1	4	0	−
LBD-7	73	Male	18	0	0	II	2	5	0	−
LBD-8	67	Female	22	0	0	III	3	5	0	−
LBD-9	64	Male	25	0	0	II	1	4	0	−
LBD-10	69	Male	23	0	0	II	1	4	0	−
CO-1	70	Male	–	0	0	II	1	0	0	−
CO-2	68	Male	–	0	0	II	1	0	0	−
CO-3	75	Male	–	0	0	II	1	0	0	−
CO-4	74	Male	–	0	0	II	3	0	0	−
CO-5	65	Male	–	0	0	II	0	0	0	−
CO-6	76	Male	–	0	0	II	1	0	0	−

Aβ = amyloid-β; AGD = argyrophilic grain disease; CAA = cerebral amyloid angiopathy; LBD = Lewy body disease; NFT = neurofibrillary tangle; PSP = progressive supranuclear palsy. + = present; − = not present.

Clinical diagnoses were made following the UK Brain Bank criteria for PD^19^ and the Movement Disorder Society-endorsed PSP study group criteria for PSP^20^: all fulfilled the clinical diagnostic criteria for Richardson syndrome. Clinical information from PSP patients was collected through prospective longitudinal assessments^21^ by two movement disorders specialists (B.C., C.F.).

### Immunostaining for CD8, microglia and protein pathology

Fifteen serial sections, each 8-μm-thick, spanning 120 μm across the midbrain were stained consecutively as follows: sections 1, 4, 7, 10, 13 for anti-p-tau in PSP and anti-α-synuclein in PD; sections 2, 5, 8, 11, 14 for HLA-DR to detect microglial cells; and sections 3, 6, 9, 12, 15 for CD8 cytotoxic T-cell marker (antibody information: Supplementary Table 1). To enable 3D-reconstruction and video demonstration (Supplementary Videos 1-3) of the distributions of p-tau (AT8) and α-synuclein (5G4) pathologies related to microglia and CD8 cells, we used three different chromogens—for AT8 and 5G4, substrate chromogen DAB: 3,3'-diaminobenzidine tetrahydrochloride (brown); for HLA-DR, magenta substrate chromogen (pink); and for CD8, substrate TMB: 3,3′-5,5′-tetramethylbenzidine dihydrochloride (blue). CD8 immunostaining labels the cytoplasm of T cells so they can be recognized unequivocally from unstained nuclei of other cell types. For details of image processing, see the Supplementary material. In PSP cases, we additionally stained for 4R- and 3R-tau. A single section was stained for AT8 in the PD cases. Sections from CO cases were also immunostained for AT8 and 5G4. Furthermore, we semi-quantitatively evaluated (0: none; 1: mild; 2: moderate; 3 severe) five brain regions in nine PSP cases and five CO (of six CO included in the study where most anatomical regions were available) for CD8 cells in parenchymal and perivascular locations.

### Cell counting

Each marker covered 40 μm through the SN. The sections were analysed using an unbiased stereological procedure. A total of 375 sections were used for analysis. For CD8, 5G4 and AT8 sections (i.e. the number of neurons with cytoplasmic deposits), a stereological counting approach was employed using Stereo Investigator software (MBF^®^). A physical fractionator probe was used with the following parameters: grid = 178 × 250 μm; probe = 60 × 60 μm; sampling grid area = 26 700 μm^2^; sampling site fraction (ssf) = 1; average number of sampling sites per case = 539. Since the total counts and density of CD8 cells were very low (many times leaving the probe empty), the approach was to extract total CD8-cell counts in the four annotated regions of the SN. We therefore calculated the true object number leading to a null sampling error (coefficient of error, CE = 0) as in previous studies.^22,23^ Perivascular CD8 cells were counted in each section and annotated region. Additionally, stereological counting of CD8 cells was performed in the MBT and red nucleus/superior cerebellar peduncle (RN/SCP) in PSP.

### Image area quantification: evaluation of microglia, total p-tau and total α-synuclein pathology load

Snapshots (×10.5 magnification) of the midbrain were imported to HALO (v2.3, Indica Labs) for quantification of HLA-DR^+^ microglia, total p-tau and α-synuclein load (for both the sum of cytoplasmic and neuropil/neurites) using the area quantification module. The same four annotations within the SN were assessed for percentage of positive tissue. The parameters were adjusted to correctly differentiate neuromelanin from pathological p-tau or α-synuclein in neuronal cytoplasm.

### Statistical analysis

Kruskal–Wallis and Mann–Whitney tests were used for categorical and non-parametric variables. Correlation between CD8-cell counts and total p-tau load and HLA-DR in PSP and between total α-synuclein load and HLA-DR in PD were analysed across different SN-subregions using Spearman’s *r*. Two-tailed Spearman’s *r* was used to analyse the association of SN-CD8-cell counts with the semiquantitative score of CD8 cells in further brain regions, as well as pathological loads. Multiple linear regression models were used to further analyse the predictors of CD8 cells in the SN, incorporating disease duration, age, microglia and pathological tissues.

## Results

### Increased number of cytotoxic T cells in PSP

**Figure 1:**
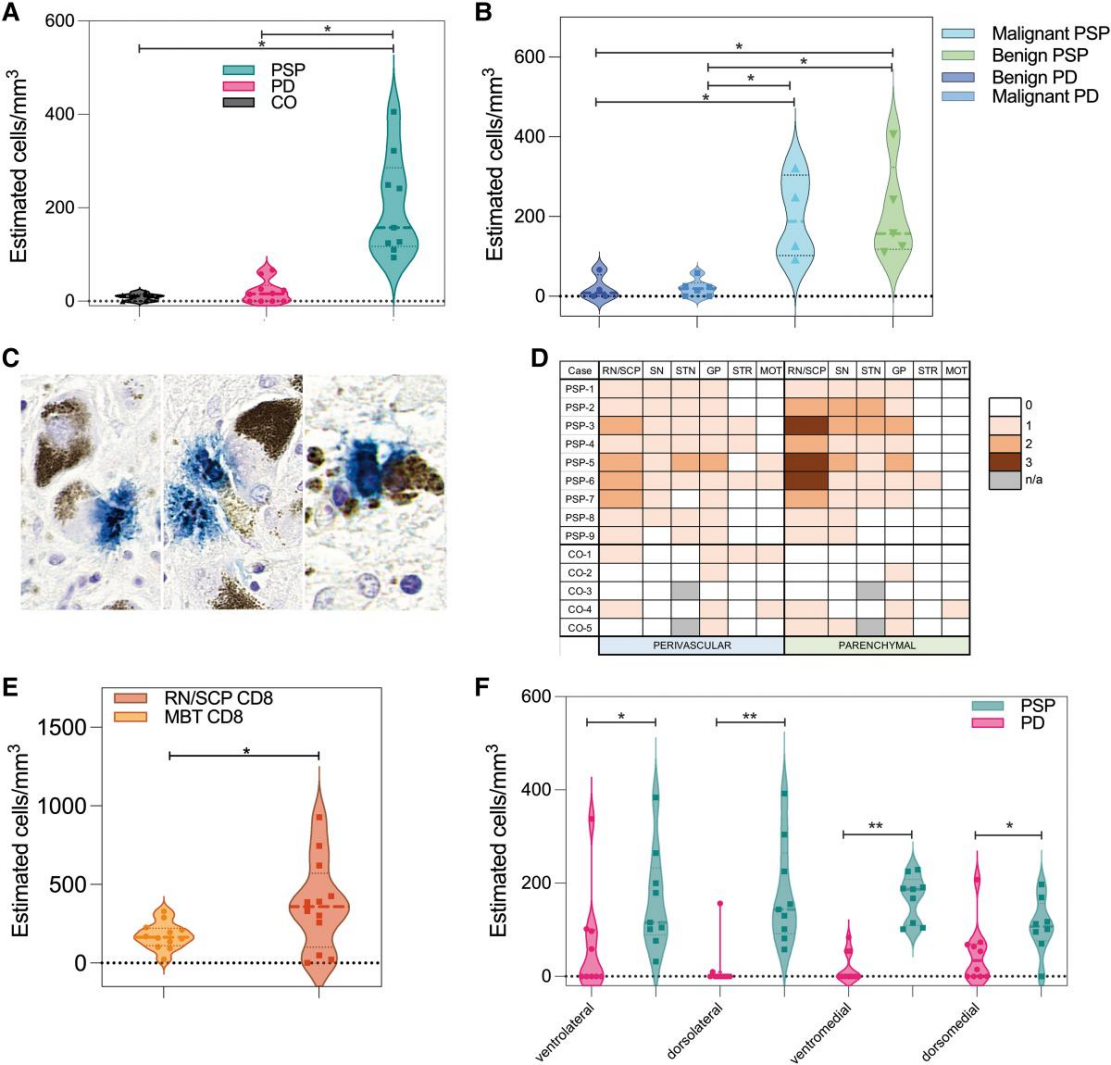
**Differences in CD8-cell counts between conditions and regions.** Density of CD8 cell infiltrates in the substantia nigra (SN) of progressive supranuclear palsy (PSP), Parkinson's disease (PD) and control (CO) subjects (**A**). CD8 cells in PSP and PD according to disease duration subgroups: benign (PSP >8 years, PD >12 years) and malignant (PSP <7 years, PD <11 years) courses (**B**). High magnification of the midbrain in case PSP-6 showing (blue-stained) CD8 infiltrates including lymphocytes in close proximity to the neuronal cell membrane (**C**). Semiquantitative scoring (0–3; n/a = not available) and heat mapping of parenchymal and perivascular CD8-cell-infiltrates in PSP-specific regions for nine PSP cases and five controls; note that only five control cases where most of the brain regions were available were included (**D**). CD8-cell count in red nucleus/superior cerebellar peduncle (RN/SCP) and midbrain tegmentum (MBT) in PSP (**E**); note that 15 samples were used for RN/SCP, since three cases had bilateral tissue available, which were all counted. CD8-cell infiltrates in the SN subregions annotated for PSP and PD (**F**). Note that in subregions, higher values than the mean of the summarized data plotted for the whole SN can be observed. STN = subthalamic nucleus; STR = striatum; GP = globus pallidus; MOT = motor cortex.

The total number of CD8 cells in the SN was significantly higher in PSP compared with PD brains [mean ± standard deviation (SD) in PSP = 203 ± 107, in PD = 20 ± 24; *P* < 0.001] and CO brains (mean ± SD = 8.81 ± 7.61; *P* < 0.001; Fig. 1A). The lowest CD8-cell count in PSP (PSP-7: 93 cells/mm^3^) was higher than the PD case with the highest CD8-cell count in the SN (LBD-3: 66 cells/mm^3^) and the highest in CO brain (CO-4: 69 cells/mm^3^). No differences were found in the number of CD8 cells between PD and CO (*P* = 0.57). We divided the PSP and the PD groups into those with a shorter disease duration (malignant-PD < 11 years, malignant-PSP < 7 years, representing a potentially more aggressive course) and those with a longer duration (benign-PD ≥ 12 years, benign-PSP ≥ 8 years, i.e. a benign course). The density of CD8 cells was higher in the SN of benign-PSP and malignant-PSP compared with malignant-PD patients (*P* = 0.0.014 and 0.009, respectively) and higher in benign-PSP than benign-PD patients (*P* = 0.01). There were no differences when comparing malignant versus benign-PD or benign-PSP (Fig. 1B).

**Figure 2:**
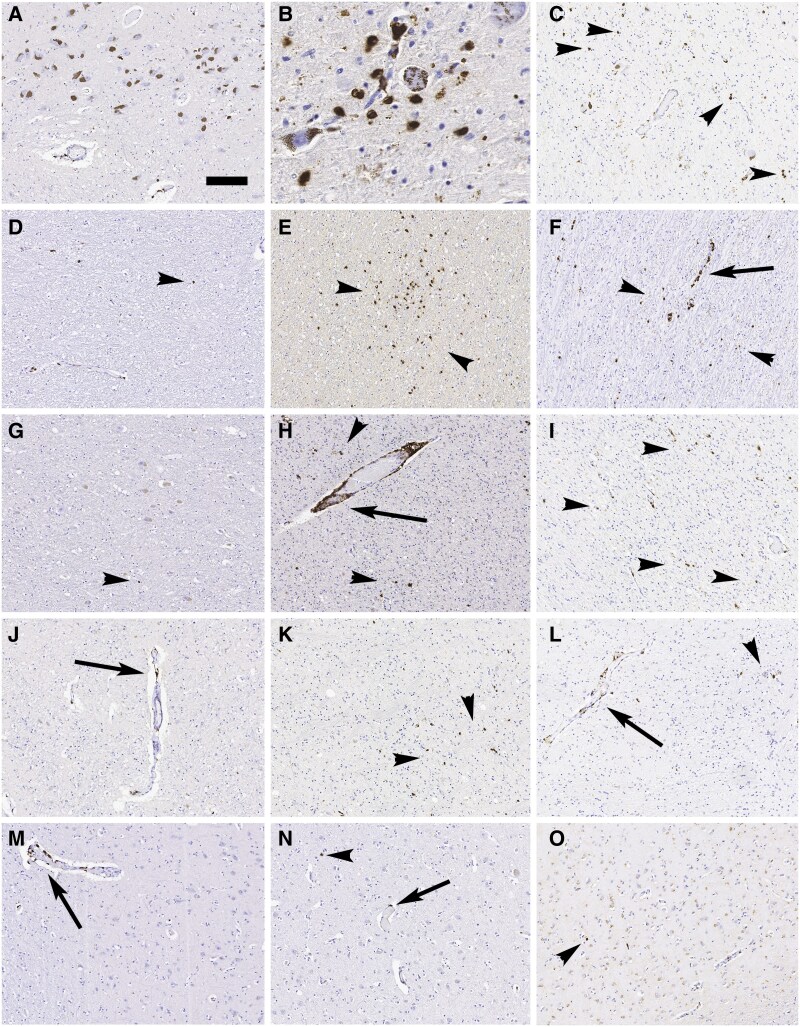
**Regional distribution of CD8-positive cells.** Immunohistochemistry for CD8-positive cells (brown) in control (CO) (**A**, **D**, **G**, **J** and **M**) and two cases with progressive supranuclear palsy (PSP) (**B**, **C**, **E**, **F**, **H**, **I**, **K**, **L**, **N** and **O**) in the substantia nigra (**A**–**C**), red nucleus/superior cerebellar peduncle (**D**–**F**), subthalamic nucleus (**G**–**I**), globus pallidus (**J**–**L**) and motor cortex (**M**–**O**). Note the more prominent CD8 cells in PSP, including CD8 cells surrounding neurons (**B**). Arrowheads indicate parenchymal and arrows perivascular CD8 cells. Scale bar in **A** = 120 μm for all images except **B**, where it represents 15 μm.

In four of nine PSP cases (PSP-3, PSP-4, PSP-5, PSP-6; Fig. 1C), we found CD8 cells and neurons in close proximity. Three of these cases had short disease duration (<7 years), and the other 8 years (PSP-6). To confirm the selectivity of the CD8 cells in PSP-affected brain regions, additional sites were stained in PSP and CO cases: globus pallidus (GP), motor cortex (MOT), striatum (caudate-putamen, STR), subthalamic nucleus (STN) and SN, as well as the RN/SCP. Comparing semiquantitative scores in these regions in PSP versus CO, only the SN, RN/SCP and STN were significantly different both for parenchymal (SN-χ^2^ = 7.98; *P* = 0.004; MBT-χ^2^ = 7.27; *P* = 0.007; STN-χ^2^ = 4.40; *P* = 0.03) and perivascular CD8-cell counts (SN-χ^2^ = 9.63; *P* = 0.01; MBT-χ^2^ = 6.43; *P* = 0.01; STN-χ^2^ = 6.40; *P* = 0.01; Figs 1D and 2). In PSP cases, the CD8-cell count was higher in the RN/SCP than in the MBT (mean ± SD for RN/SCP = 409 ± 264, and MBT = 201 ± 78; *P* = 0.02; Fig. 1E).

CD8-cell counts were not significantly different in PSP-annotated subregions. Group comparisons between PSP, PD and subregions were all significantly higher for PSP (Fig. 1F).

### Image quantification of microglial load

**Figure 3:**
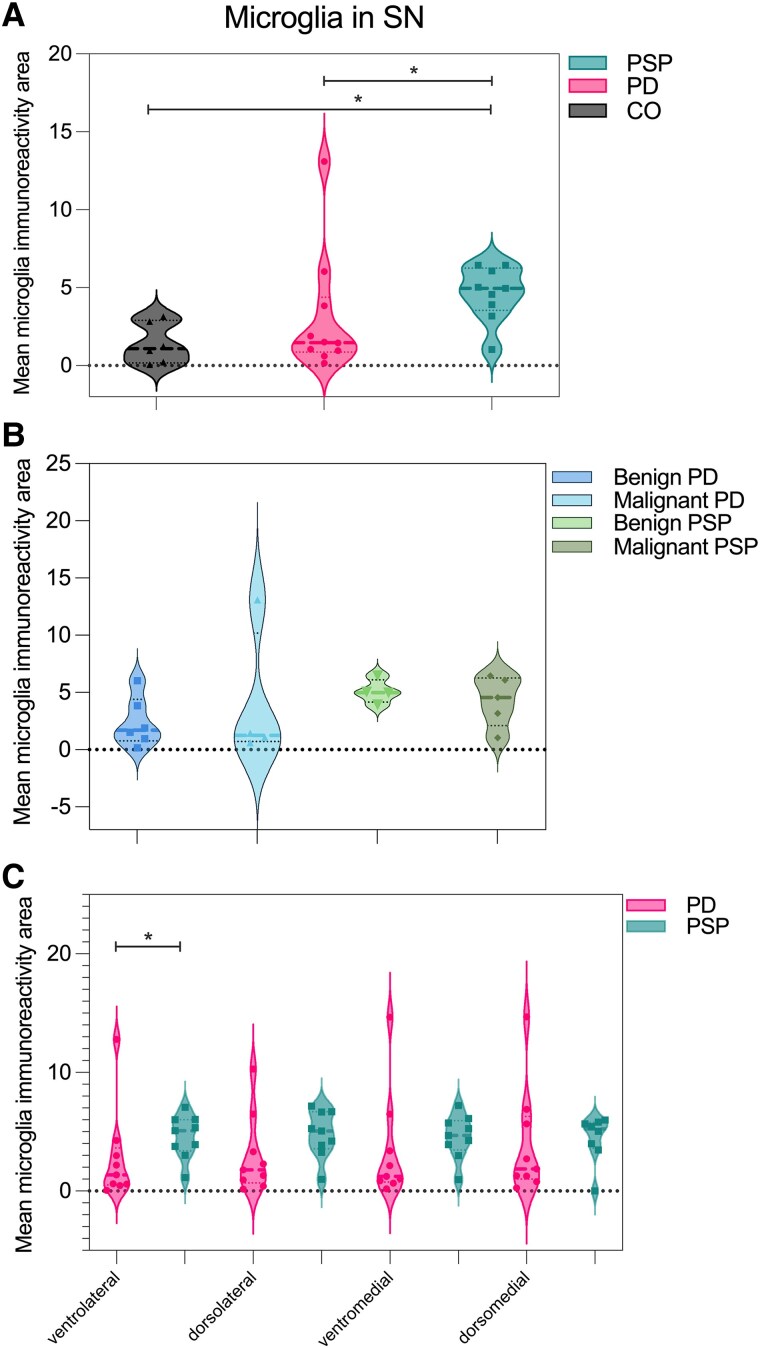
**Microglial load under different conditions.** Mean HLA-DR (microglia) immunoreactivity area in the substantia nigra (SN) of progressive supranuclear palsy (PSP), Parkinson's disease (PD) and control (CO) samples (**A**). Mean microglia immunoreactivity area in SN according to disease duration subgroups: benign (PSP >8 years, PD >12 years) and malignant (PSP <7 years, PD <11 years) (**B**). Mean microglia immunoreactivity area in SN subregions of PSP and PD, for microglia detection (**C**). Note that in subregions, higher values than the mean of the summarized data plotted for the whole SN can be observed.

The mean area of microglia immunoreactivity was higher in PSP than PD (4.62% ± 1.74% versus 3.06% ± 3.9%; *P* = 0.042) and in PSP compared with CO (1.40% ± 1.30%; *P* = 0.006) but not PD compared with CO (*P* = 0.309; Fig. 3A). There were no differences between benign and malignant-PSP or between benign and malignant-PD (Fig. 3B). In PSP, the area of microglia immunoreactivity was similar between subregions. A significant difference in the area of microglia immunoreactivity was found between PSP and PD in the ventrolateral SN (*P* = 0.03; Fig. 3C).

### P-tau and α-synuclein pathology in the substantia nigra in PSP and PD

The density of cells containing neurofibrillary tangles (NFT) in PSP was 251 ± 133 cells/mm^3^, whereas that of Lewy body-containing cells in PD was 117 ± 59 cells/mm^3^. In PSP brains, anti-4R-tau staining was homogeneous and marked throughout the whole midbrain, whereas 3R-tau-positive cells were absent in the SN, with only single immunoreactive cells in the dorsal raphe nucleus, as is frequently seen with ageing. In PD brains, there were no p-tau-immunoreactive neurons throughout the midbrain or SN, except for two cases that had AD co-pathology (LBD-1 and LBD-7) and another case with primary ageing-related tauopathy (PART, LBD-3; Table 1).^3^ The mean area of p-tau load detected in PSP was 22.52% ± 9.54, and the mean area of pathological-α-synuclein load in PD was 12.56% ± 5.90. The p-tau load in PD SN was 1.16% ± 1.80.

The stereological counts of p-tau cells (NFT) in PSP showed variability among SN subregions, but this did not reach statistical significance overall (ventromedial SN, 221 ± 156 cells/mm^3^; dorsomedial SN 333 ± 281 cells/mm^3^; ventrolateral SN 224 ± 127 cells/mm^3^; and dorsolateral SN 245 ± 171 cells/mm^3^). The count of cells containing NFTs was not associated with CD8-cell counts in SN subregions. The number of neurons showing α-synuclein pathology in PD showed variability between SN subregions (ventromedial SN: 188 ± 147 cells/mm^3^; dorsomedial SN: 60 ± 68 cells/mm^3^; ventrolateral SN: 142 ± 111 cells/mm^3^; and dorsolateral SN: 78 ± 89 cells/mm^3^) but were not significantly different among these subregions.

In PSP, the p-tau load also showed variability among SN-subregions, without regional differences (ventromedial SN 0.83% ± 0.33% area; dorsomedial SN 0.96% ± 0.27% area; ventrolateral SN 1.11% ± 0.45% area; dorsolateral SN 0.83% ± 0.52% area). In PD, the α-synuclein load was significantly higher in the ventromedial SN than the dorsolateral and dorsomedial SN (*P* = 0.006 and *P* = 0.008, respectively). The extent of protein-pathology-affected tissue was higher for PSP than PD in the dorsolateral, ventrolateral and dorsomedial SN (*P* = 0.01, *P* = 0.01 and *P* = 0.001, respectively; data not shown). In PD brains, there was no association between CD8-infiltrates and the density of neurons showing α-synuclein-pathology in the different subregions.

### Correlation between variables

In PSP, age at death correlated with microglial-load (*r* = 0.90, *P* = 0.001) but not with other variables. No other variables showed correlations in either PSP or PD. Multiple regression analysis did not reveal any effect of variables on CD8 counts.

## Discussion

We observed a consistent cytotoxic T-cell response in early affected brain regions in PSP, far more than in PD. Our study using a stereological approach confirmed the findings of a recent study.^24^ Expanding beyond that study, we have shown that the midbrain is more affected by CD8 cells than cortical regions and demonstrated the close proximity of CD8 cells to SN neurons in PSP. On the other hand, areas beyond the midbrain and STN did not show mentionable numbers of CD8 cells, suggesting that the primary target of the CD8 cells is found in the midbrain, reminiscent of postencephalitic parkinsonism.^25^ However, in contrast to postencephalitic parkinsonism, there is a lack of plasma cells.^26^

The potential role of cellular adaptive immunity in PSP is highlighted by evidence of higher lymphocytes in cases of FTLD-tau.^12^ That report found elevated CD4 cells but not CD8 cells in FTLD brains, and correlation of CD8 cells with p-tau; however, they evaluated frontal cortex, which is typically not the earliest affected region.^1^ We found CD8 cells in close proximity to surviving neurons in four PSP brains. This phenomenon is an important hallmark of immune-mediated brain disorders, even without prominent inflammatory cell infiltration as in infectious encephalitides.^9^ This observation raises the possibility that autoimmune pathomechanisms might be present in a subset of PSP cases. This would suggest that diverse aetiologies could contribute to the common histopathological hallmarks summarized currently as PSP-type pathology.

As a limitation, we did not perform screening for IgLON5 or other autoimmune encephalitis-related antibodies. However, we did not find the typical clinical symptoms or the constellation of 3R- and 4R-tau immunoreactive pathology as reported in IgLON5 disease in our cases.^27^ In another study where we evaluated the HLA-locus in our PSP cohort, we did not find the HLA haplotypes reported to be associated with LGI1 (HLA-DRB1*07:01) or CASPR2 (HLA-DRB1*11:01) antibody-related diseases, except for one case in our study with unequivocal 4R-positive PSP-type tau pathology (PSP Case 5), which showed the IgLON5 disease-related DRB1*10:01-DQB1*05:01 haplotype.^28^ Four-repeat predominant tau pathology is rarely reported in IgLON5 disease.^27^ Since we found elevated CD8 cells in PSP cases without this haplotype, our observations support the notion that some cases considered as typical PSP (both at clinical and neuropathological levels) could have an autoimmune pathogenesis.

A previous study in human brains found greater CD8-cell infiltration in PD brains compared with controls,^13^ which contrasts with our findings using a stereological approach, where no difference was seen. Our observations are different also from a recent study.^24^ Apart from our stereology approach, which was not used in the previous studies,^13,24^ the different results highlight the underappreciated fact that Lewy body pathology (i.e. PD) cases might have various aetiologies and a spectrum of mixed pathologies contributing to the discrepancies. Interestingly, in the previous study,^13^ the most robust infiltration was seen in the earliest stages of disease prior to neuronal loss, and a much milder infiltration was observed in the next stage when neuronal loss occurred. This may also explain some of the discordance with our findings. Nevertheless, in our PSP cases the number of CD8 cells exceeded that in PD, suggesting that the mechanisms leading to a cytotoxic T-cell response might be very different in the two disorders.

Finally, p-tau pathology was absent in our PD cohort, contrasting with a recent report,^29^ which included patients with mild parkinsonian signs and hypothesized that tau accumulation may disappear as the disease progresses. Importantly, subcortical nuclei can be affected in primary age-related tauopathy and AD as well; however, in AD a decrease in striatal dopamine transporter was reported that did not correlate with the tangle counts in the SN.^30^

Limitations of this research are selection bias and small sample size. The sample size is limited due to the rarity of PSP, and selection bias is an inevitable feature of brain bank studies, given the design within specialized academic centres of referral. Grouping the cases into benign and malignant forms might be arbitrary, and the correlation between duration of illness and inflammatory markers should be evaluated in larger cohorts. These limitations should be considered when interpreting the results, which will require further confirmatory studies.

## Conclusion

Our study demonstrates cytotoxic T cells predominating in the midbrain of PSP brains beyond that seen in PD. We propose that a subset of PSP cases may have an autoimmune pathophysiological component that might require further investigation into pathological subtyping and be considered in stratification for new therapeutic targets.

## Supplementary Material

awaf135_Supplementary_Data

## Data Availability

Upon request from qualified researchers, data will be made available.
