# Supplementary material for: Midbrain cytotoxic T cells as a distinct neuropathological feature of progressive supranuclear palsy
Source: Brain. 2025 Apr 15;148(8):2650–7. doi: 10.1093/brain/awaf135 (PMC12316005; doi:10.1093/brain/awaf135)

Supplementary material

Methodology

Stereological procedure

In PSP (*n*=9), PD (*n*=10) and controls (*n*=6), 70-micron thickness of SN were stained for: CD8, microglia (HLA-DR), tau (AT8), and synuclein (5G4).

Resulting in 5 serial sections (same stain every 21 μm) suitable for stereological analyses.

Additional counting: RGB analyses of area (μm^2) of HLA, tau (AT8), and synuclein (5G4) with HALO software.

For AT8-sections (in PSP) and 5G4-sections (in PD), the same probe and parameters were used within the same annotations in the SN to count for neurofibrillary tangles in PSP (cytoplasmic AT8 immunoreactive structures in neurons) and neurons with Lewy-bodies- and other 5G4 immunoreactive fibrillar and dot-like deposits in PD (cytoplasmic 5G4 in neurons). These resulted in an average CE = 0.28. For brainstem sections of CO, the same probe and parameters were used within the same annotations in the SN to count for neuromelanin-containing pyramidal neurons except for ssf=0.84; average number of sampling sites per case (5 sections) =1235; and CE= 0.056.

Results

Density of neuromelanin-containing cells in the SN of CO brains

In CO brains, SN count of neuromelanin-containing pyramidal neurons resulted in a density of 918 cells/mm^3.

Supplementary Table 1. Characteristics of the primary antibodies.

**Antibody**	**Company, location**	**Dilution**	**Target**
AT8, MN1020	Thermo Fisher, Waltham, MA, US	1:1000	Phosphorylated tau
5G4	Roboscreen, Leipzig, Germany	1:4000	α-synuclein
CR3/43	Agilent/Dako, Santa Clara, CA, US	1:200	HLA-DR microglia
M7103	Agilent/Dako, Santa Clara, CA, US	1:200	CD8 cells
05-803	Millipore/Sigma, Darmstadt, Germany	1:200	3-repeat tau
05-804	Millipore/Sigma, Darmstadt, Germany	1:200	4-repeat tau

Supplementary figures legends

Supplementary figure 1. The stereological procedure

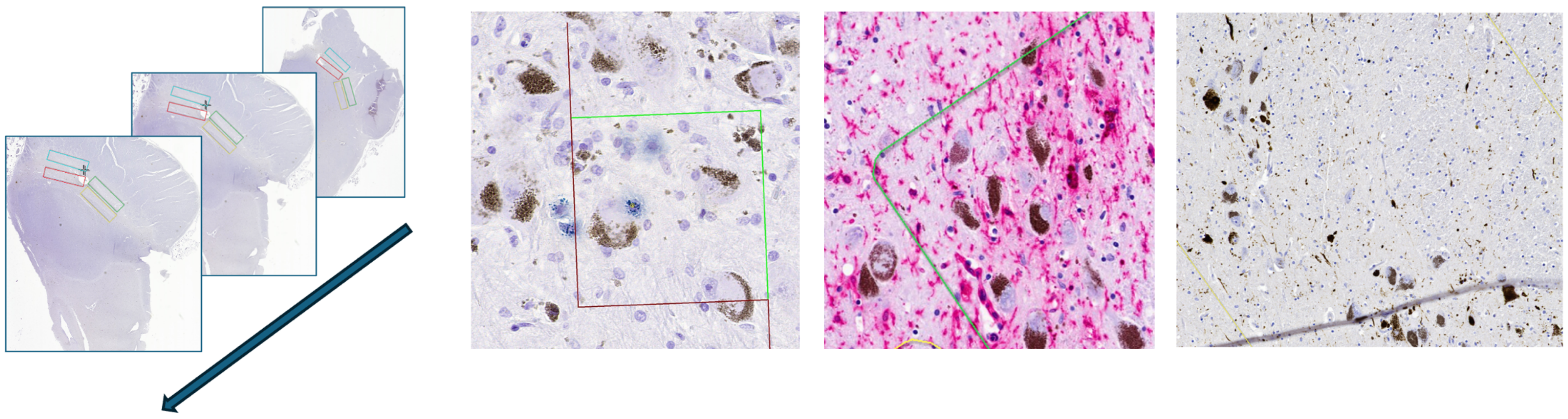

Supplementary figure 2. Examples of close apposition of T-cells to neuromelanin containing cells in three PSP cases.

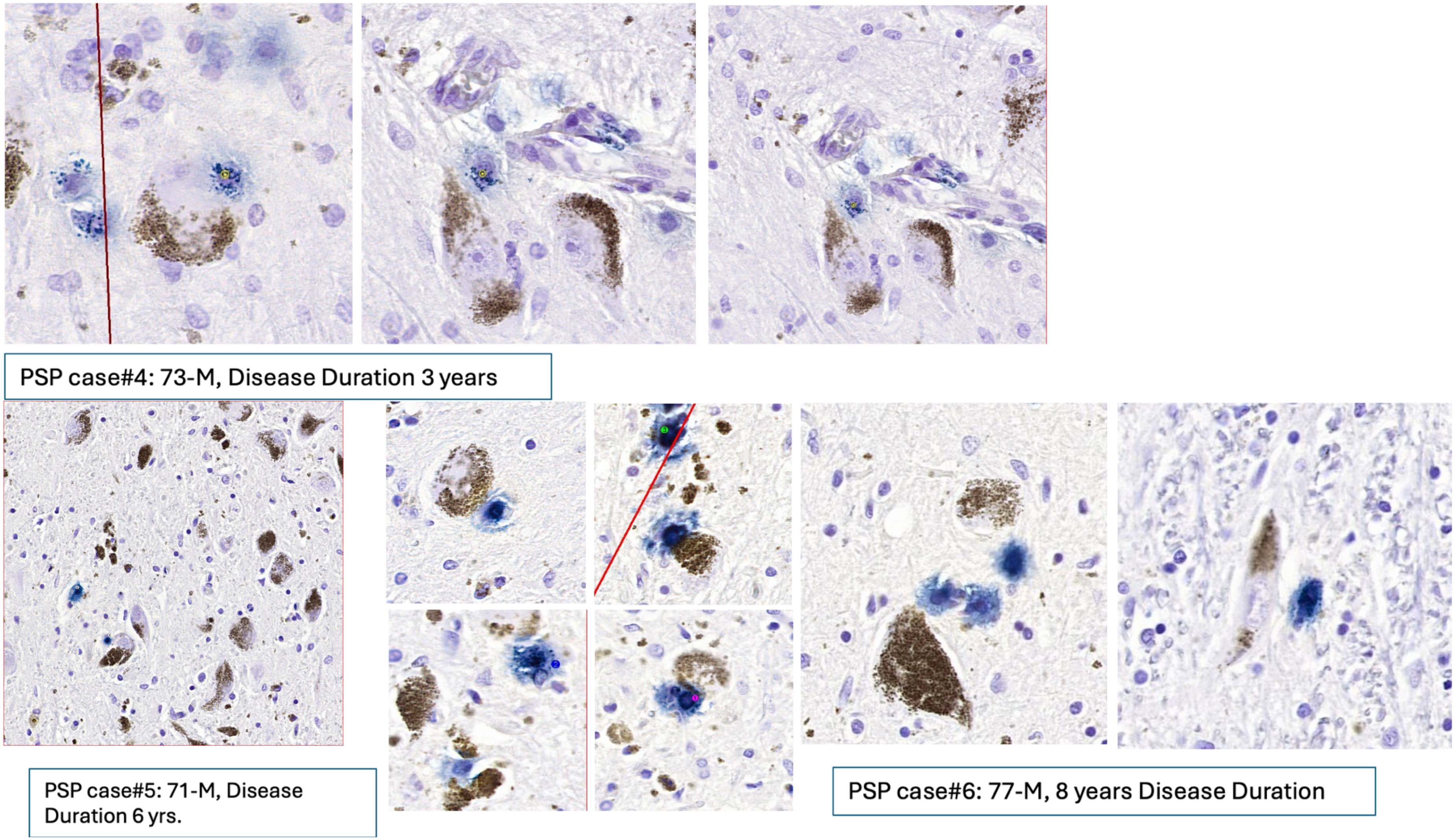

Supplement: awaf135_Supplementary_Data [file awaf135_supplementary_data.zip › brain-2024-02625-File006.pdf]
