# Supplementary material for: Midbrain cytotoxic T cells as a distinct neuropathological feature of progressive supranuclear palsy
Source: Brain. 2025 Apr 15;148(8):2650–7. doi: 10.1093/brain/awaf135 (PMC12316005; doi:10.1093/brain/awaf135)
Supplement: awaf135_Supplementary_Data [file awaf135_supplementary_data.zip › brain-2024-02625-File010.pdf]

# Author Contributions

Concept and design: BC, SLF, CF, GGK

Acquisition, analysis, or interpretation of data: BC, SLF, CF, SL, SK, JL, SHF, MCT, AEL, GGK

Drafting of the manuscript: BC, SLF, GGK

Critical review of the manuscript for important intellectual content: All authors.

Statistical analysis: BC

Obtained funding: AEL, GGK

Administrative, technical, or material support: BC, SLF, CF, SL, SK, JL, SHF, MCT, AEL, GGK

Supervision: SLF, GGK
